# Diet of Eastern Imperial Eagle (*Aquilaheliaca*) in Bulgaria: composition, distribution and variation

**DOI:** 10.3897/BDJ.10.e77746

**Published:** 2022-01-10

**Authors:** Dimitar Demerdzhiev, Zlatozar Boev, Dobromir Dobrev, Nikolay Terziev, Nedko Nedyalkov, Stoycho Stoychev, Tseno Petrov

**Affiliations:** 1 Bulgarian Society for the Protection of Birds, Sofia, Bulgaria Bulgarian Society for the Protection of Birds Sofia Bulgaria; 2 National Museum of Natural History, Sofia, Bulgaria National Museum of Natural History Sofia Bulgaria

**Keywords:** top predator, food spectrum, diet, raptors, long-term studies, population

## Abstract

The Eastern Imperial Eagle (EIE) is a top predator exploiting different prey in different parts of its distribution. In this study, we summarise data collected over a long period of time (for 25 consecutive years), identifying key prey species in the different regions, as well as clarifying seasonal preferences in the eagle’s diet. Most studies on the EIE food composition covering different parts of the species distribution range analyse the breeding season, while data about the winter diet are scarce. To the best of our knowledge, this is the first study detailing the differences in EIE’s dietary preferences between the breeding and the winter periods. We identified 4891 specimens belonging to 196 different taxa, which represents the most comprehensive study considering the diet diversity of this threatened species. Mammals represented the largest proportion of the diet, followed by birds and reptiles. Northern White-breasted Hedgehog was the most common prey, accounting for 25.7% of the total prey caught and 26.75% of the biomass. The European Souslik was the second most important prey with 14.35% participation in the eagle’s diet, but with a 3.75% contribution to the biomass. As we predicted, prey composition and main prey species varied spatially and seasonally. Modelling differences in the EIE diet, we found that the “territory effect” had the strongest impact on the dietary variations. Diet diversity differed significantly between regions (F = 12.6, df = 4, p = 0.01). During the breeding season, eagles fed mainly on Hedgehogs (29.88%), Sousliks (16.85%) and Storks (7.74%), while the winter diet was predominantly small rodents (44.17%) and songbirds (13.96%). We found that top predators, such as EIE, have successfully adapted to a novel food source, which is abundant in the area. The detected flexibility in the diet of the species and its ability to switch to alternative prey, if available, when the primary prey decreased, should be considered when planning species conservation efforts. Investigating the temporal change of the main prey in the eagle’s diet is also crucial for further species conservation measures.

## Introduction

The Eastern Imperial Eagle (*Aquilaheliaca*), hereafter (EIE), is a large-size raptor species breeding from Central Europe, the Balkans, Central Asia and South Siberia to China and Mongolia (BirdLifeInternational 2021). While the European population is considered stable (Demerdzhiev et al. 2011), the species is classified as globally vulnerable and decreasing (BirdLifeInternational 2021). After 2000, following a severe decline during the second half of the 20^th^ century, gradual population recovery in Bulgaria has been recorded (Demerdzhiev et al. 2014a, Demerdzhiev et al. 2015), reaching 41 occupied territories in 2021.

The EIE is a top predator exploiting different prey in different parts of the distribution area (Cramp and Simmons 1980, del Hoyo et al. 1994). While Sousliks (*Spermophilus* sp.) are predominant for the largest Eastern populations of Russia and Kazakhstan (Belik et al. 2002, Karyakin et al. 2008, Karyakin et al. 2011, Katzner et al. 2006), in the other parts of the species range, the diet of the EIE consists of different-sized mammals, birds and reptiles. However, in the Carpathian Basin, the main prey species are European Hare (*Lepuseuropaeus*), European Hamster (*Cricetuscricetus*) and Common Pheasant (*Phasianuscolchicus*) (Chavko et al. 2007, Horváth et al. 2010, Horváth et al. 2018, Horal 2011, Wichmann 2011). The EIEs breeding in European Turkey feed mainly on Northern White-breasted Hedgehog (*Erinaceusroumanicus*), tortoises – Hermann's tortoise (*Testudohermanni*) and Greek tortoise (*Testudograeca*) and Yellow-legged Gull (*Larusmichahellis*) (Demerdzhiev et al. 2014b), while in the Caucasus, reptiles, hedgehogs (*Erinaceus* spp.) and small rodents are predominant in the diet (Abuladze 1996, Horváth et al. 2011).

Sparse data about the diet of the species in Bulgaria were published in the works of Hristovich (1890), Klein (1909), Zhelev et al. (2009) and Stoychev et al. (2012). Partial regional studies on the species food spectrum were provided for the Sredna Gora Mnt. (Simeonov and Petrov 1980), the Sakar Mnt. and the Dervent Heights (Marin et al. 2004). For the Sredna Gora Mnt., the European Souslik (*Spermophiluscitellus*) was identified as a major food source (Simeonov and Petrov 1980), while the most common prey for the Sakar Mnt. was the Northern White-breasted Hedgehog and for the Dervent Heights – the European Hare (Marin et al. 2004).

In this study, we summarise data collected over 25 consecutive years, identifying the key prey species in the different regions, as well as clarifying the seasonal preferences in the eagle’s diet. Most of the studies on the EIE food composition covering different parts of the species distribution range analyse the breeding season (Katzner et al. 2005, Katzner et al. 2005, Karyakin et al. 2008, Karyakin et al. 2011, Horváth et al. 2010, Horváth et al. 2018, Demerdzhiev et al. 2014b), while data about the winter diet are scarce (Zhelev et al. 2009, Demerdzhiev 2011, Hasani et al. 2012). To the best of our knowledge, this is the first study detailing the differences in EIE’s dietary preferences between the breeding and the winter periods. We predicted that prey composition and main prey species varied amongst geographical regions and seasons.

## Methods

### Study area

**Figure 1.**
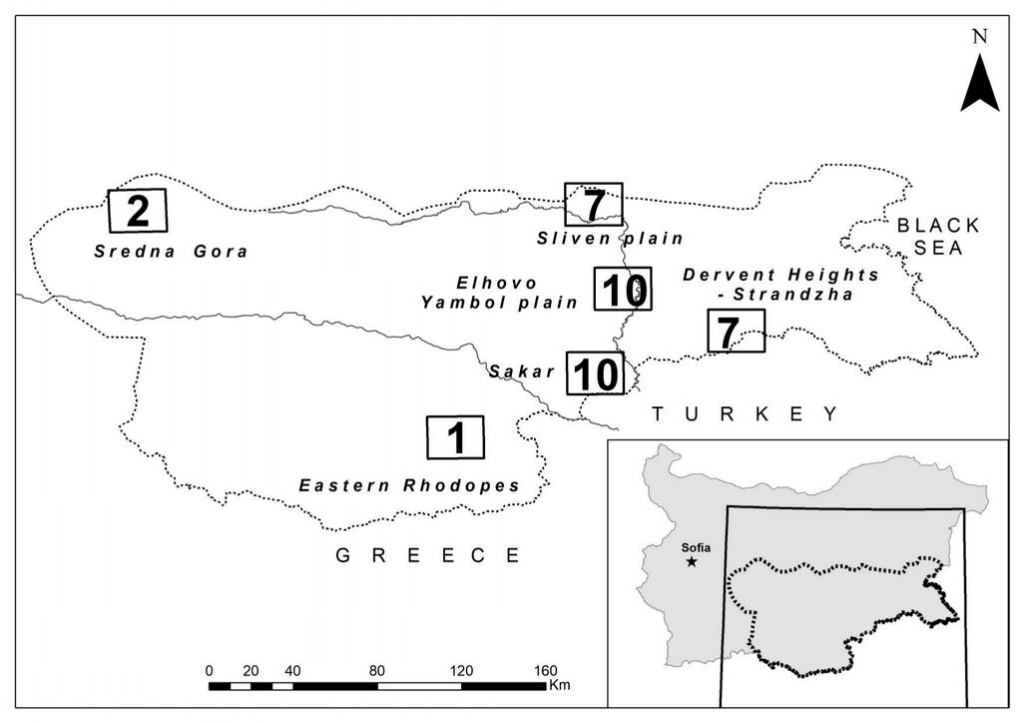
Map of the studied nests.

We collected dietary data from eagles during the period 1996-2020 from 37 breeding sites distributed in six regions (Table 1) (Fig. 1). In the mountainous habitats (340-1100 m a.s.l.) in the SG and the ER, the eagles breed on Sessile oak (*Quercuspetraea*) and Scots pine (*Pinussilvestris*). The species mountain habitats are identical, characterised by small open spaces (pastures and meadows) and considerable forest cover (Demerdzhiev 2011). The EIEs in other regions of south-east Bulgaria occupy hills and lowlands, where pastures, meadows, agricultural fields and small forest patches form habitats of different structure. They build their nests on single trees or in small groups of trees, mainly hybrid poplars (*Populus* sp.) or Hungarian oak (*Quercusfrainetto*), Downy oak (*Quercuspubescens*) and Turkey oak (*Quercuscerris*), often along small streams or in ravines. The habitats in the Sakar Mnt. and the DHWstr. are characterised by pastures, meadows, at times overgrown with shrub formations of Oriental hornbeam (*Carpinusorientalis*) and Christ's thorn (*Paliurusspina-christi*) with xerothermal grass formations (Bondev 1991). The habitats in the SP and the EYP consist of intensive agricultural fields and smaller grassland patches without forests.

### Data collecting

Each nesting site was visited twice in each of the following periods: November-February, June-August (post-fledging period). Food remains, bones, feathers and pellets were collected inside and under nests and roosts (Katzner et al. 2005). The following type of remains were not included in the data in order to reduce the bias of indirect sampling, even if they were found under the nest sites or roosting trees: (1) single feathers, which could be shed by live birds; (2) full carcasses of large animals, which could not be brought there by the eagles; (3) old or deteriorated samples, which could have remained from previous years (Horváth et al. 2018). A total of 2471 pellets and 10780 bones and bone fragments were collected. This material was identified through the comparative osteological collections of the National Museum of Natural History at the Bulgarian Academy of Sciences. Whenever possible, the minimum number of individuals (MNI) in each pellet or prey remains was estimated, based on the number of skeletal or keratinised body parts (Katzner et al. 2005). The MNI was determined by taking into account the age (juvenis, subadultus, adultus), the sex and the size differences between individuals.

### Data analyses

The materials collected from 1 June to 31 August were referred to the eagles’ breeding season and those from 1 November to 1 March to their autumn-winter diet. The body mass of the specimens of the various species was determined by Petrov (1964), Simeonov and Petrov (1980), Boev (1986), Simeonov et al. (1990), Böhme (1993), Kunstmüller (2000), Dunning (2008) and Aulagner et al. (2009). An average body mass was given, calculated on the basis of the average mass of individual specimens. When the material was identified up to genus level, the average values for the presented species of the genus were given. The carrion biomass was not taken into account.

In order to identify the diet differences between regions and seasons, the prey items were grouped into the following main categories, based on their specific ecological requirements: Lizards & Snakes (Squamata), Tortoises (Testudines), Water birds (Anatidae, Ardeidae), Poultry (Gallusgallusf.domestica, Anseranserf.domestica, Meleagrisgallopavof.domestica, Pavocristatusf.domestica), Phasianids (Phasianidae), Gulls (Laridae), Doves (Columbidae, Feral Pigeon), Song birds (Non-Corvidae Passerines), Corvids (Corvidae), Stork (*Ciconiaciconia*), Raptors & Owls (Accipitridae, Falconidae, Strigidae, Tytonidae), Hedgehog, Hare, Souslik, Rodents (Rodentia excl. European Souslik), Carnivores (Carnivora), Carrion (Artiodactyla, Perissodactyla) and Other Animals.

To understand differences in diets amongst regions, we used Generalised Linear Mixed Models (GLMM) with Poisson distribution and Log link function. Our response variable was region and our predictors were food categories that showed high Likelihood Score (p ≤ 0.07) in the likelihood estimation (Table 2). We ran our “Global model” in two scales: first with prey items and second with biomass adjusted data as a predictor variable. We merged territories from SG (n = 2) and ER (n = 1) into a group of high mountain regions (HM) due to the small sample sizes and the similar habitat conditions (Demerdzhiev 2011). “Eagle territory” was included in the models as a random effect. To determine which diet factors would affect the region differences, we used Akaike Information Criterion, corrected for small sample sizes (AICc), for model selection and chose the models with the lowest AIC_c_ value from the set of our candidate models. All models with an AIC_c_ value < 2 from the model with the lowest AIC_c_ (AIC_cmin_) were considered best models (∆AIC_c_ = AIC*_i_*– AIC_cmin_) (Burnham and Anderson 2002).

The relative importance of each model was estimated through the weight of AICc (*w*), so that all the weights for all models added up to 1. We also used explanatory parameter estimates with Lower (95%) and Upper CL (95%) and a probability value (p) of the explanatory factors.

To find out the diet differences between seasons, we used the non-parametric Mann-Whitney U Test with continuity correction.

All data were analysed using Statistica for Windows, Release 12 (StatSoft Inc 2013), R v.2.15.2 (R Core Team 2012) and Past Version 3.14 (Hammer et al. 2001). Results with p ≤ 0.05 were considered significant. Values were provided as means ± SE.

## Results

### Diet diversity

We identified 4891 specimens belonging to 196 different taxa (Suppl. material 1). Mammals represented the largest proportion of the diet (64.34% of individuals; 56.13% of biomass), followed by birds (25.5% of individuals; 36% biomass) and reptiles (8.81% presence; 7.14% biomass). Amphibians, fishes and insects had less than 2% participation in the eagle’s diet. Of all identified preys, the greatest diversity was found in birds, at least 109 taxa (ca. 25% of the country’s avifauna), followed by mammals (n = 54 taxa, ca. 50% of the country’s mammalian fauna), reptiles (n = 17 taxa, ca. 50% of the country’s herpetofauna), fish (n = 9 taxa), insects (n = 4 taxa) and amphibians (n = 2 taxa). One species of crab was also found in the eagle’s diet. Northern White-breasted Hedgehog was the most common prey, accounting for 25.7% of the total prey caught and 26.75% of the biomass. The European Souslik was the second most important prey with 14.35% participation in the eagle’s diet, but with 3.75% contribution to the biomass. The Common Vole (*Microtusarvalis*) accounted for 7.52% of the prey items, but less than 0.5% of the biomass, followed by the White Stork with 6.42% participation and 21.73% contribution to the biomass and the European Hare (5.46% of the victims; 16.41% of the biomass).

### Regional differences in the diet

Modelling differences in the EIE diet, we found that “random effect” had the strongest impact on the dietary variations (Table 3). This factor determined the first-ranked model both with regard to identified prey items (ΔAIC = 0.00, *w* = 0.46) and biomass contribution (ΔAIC = 0.00, *w* = 0.57). Presence of Northern White-breasted Hedgehog, Tortoises, Lizards & Snakes and biomass from Lizards & Snakes and Rodents shaped regional differences of eagle’s diet (Table 3). However, territories from Sakar Mnt. had a more powerful effect on dietary differences (*β* = 0.12 ± 0.05, p = 0.01) (Table 3).

**Figure 2.**
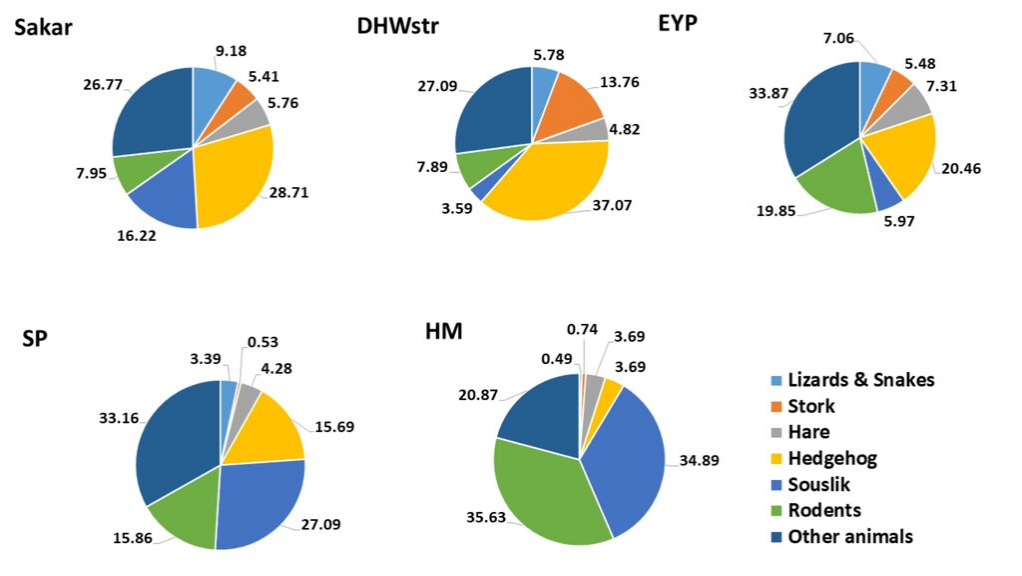
Distribution of the main food components of EIE diet in different regions (HM includes Sredna Gora Mnt. and Eastern Rhodope Mnt.).

Diet diversity differed significantly between regions (F = 12.6, df = 4, p = 0.01), being higher in the EYP (*Hʹ* = 3.488, n = 821) and the Sakar Mnt. (*Hʹ* = 3.119, n = 1961) and lower in the SG & ER (*Hʹ* = 2.516, n = 407). Regional differences were due to a much lower proportion of Lizards & Snakes in the SG (0.56%) and the SP (3.39%) and higher in the Sakar Mnt. (9.18%) and EYP (7.06%) (Fig. 2). Hedgehog was a common prey for eagles in the DHWstr. (37.07%) and the Sakar Mnt. (28.71%) and rare in the SP (15.69%) and high mountains (ER: 4.17%; SG: 3.62%). In contrast, eagles in the SG exploited mainly Sousliks (38.44%) and other rodents (39.27%), while in the DHWstr. (3.59%) and the EYP (5.97%), the Sousliks share in the diet was lower (Fig. 2). Eagles from DHWstr. exploited tortoises as an additional prey (4.03%) much more than those breeding in SG (0.28%) and EYP (1.58%). However, tortoises were practically not present in the eagle diet from SP. White Stork was common prey for eagles breeding in DHWstr. (13.76%) and very rare for territories in HM (0.74%) and SP (0.53%).

### Seasonal differences in the diet

**Figure 3.**
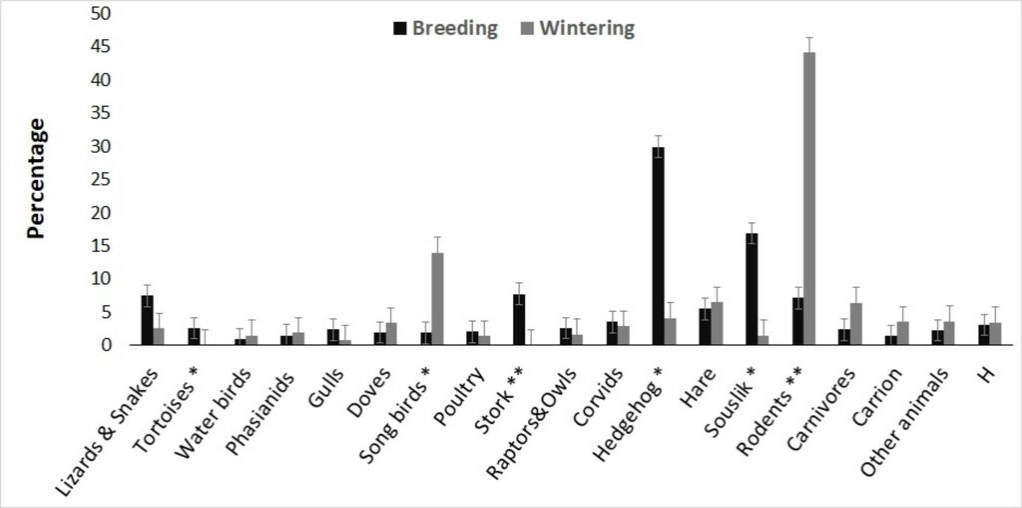
Proportion of different food categories in different seasons. Significant values are marked with: * p ≤ 0.05; ** p ≤ 0.01.

The seasonal differences in the EIE diet were determined considering six major food components (Fig. 3). During the breeding season (n = 4096 preys), eagles fed mainly on Hedgehogs (29.88%), Sousliks (16.85%) and Storks (7.74%). Tortoises also showed significant seasonal differences (Z = 1.98, p = 0.05). The winter diet (n = 795 prey) included exclusively small rodents (44.17%) and songbirds (13.96%). The proportion of Carnivores was greater in the winter period (6.42%), although the differences when compared to the summer (2.39%) were not significant (Z = 1.88, p = 0.06). The consumption of carrion by eagles in winter (3.52%) was greater than in the breeding season (1.46%), but there was no trend (Z = 1.46, p = 0.14). The diet diversity index in winter (*Hʹ* = 3.474) was also larger than the mean value in the breeding season (*Hʹ* = 3.063) (Fig. 3).

## Discussion

The great diversity of species in the food spectrum of the EIE proved its opportunism towards feeding. The identification of nearly two hundred different taxa of victims in our study supported the hypothesis of successful adaptation of the EIE to food sources (Katzner et al. 2005, Demerdzhiev et al. 2014b, Horváth et al. 2018). This was probably related to the habitat heterogeneity in Bulgaria since more diverse habitats would mean more and diverse prey (Penteriani et al. 2002). However, this issue needs further clarification.

Mammals were the most common group of vertebrates in the EIE’s diet in our study, as well as in previous studies (Katzner et al. 2006, Karyakin et al. 2008, Karyakin et al. 2011, Horváth et al. 2010, Horváth et al. 2018, Demerdzhiev et al. 2014b), but the species can exploit various animals in the different parts of its distribution range (Abuladze 1996, Turchin and Sobolev 1996, Karyakin 1999, Belik et al. 2002, Sorokin 2009, Horváth et al. 2011). Our data demonstrated that four prey species – Northern White-breasted Hedgehog, European Souslik, White Stork and European Hare - were the most important prey in the diet of the EIE in Bulgaria. Hedgehogs (*Erinaceus* sp.) were rarely found in the eagle’s diet in the Pannonian population (Chavko et al. 2007, Horváth et al. 2010, Horváth et al. 2018) and in the large eastern population in Russia and Kazakhstan (Lobachev 1960, Varshavskii 1973, Karyakin 1999, Bragin 2000, Katzner et al. 2006). However, hedgehogs were amongst the main prey species of the eagles in European Turkey (Demerdzhiev et al. 2014b) and Caucasus (Horváth et al. 2011). Storks were found as victims of pairs distributed in European Turkey, but in a lower proportion (Demerdzhiev et al. 2014b). The European Hare was the most important prey in the EIE diet in the Carpathian Basin (Chavko et al. 2007, Horváth et al. 2010, Horváth et al. 2018, Horal 2011, Wichmann 2011). Sousliks were an essential food source for EIE breeding in the vast areas of Russia and Kazakhstan (Varshavskii 1973, Solomatin 1974, Belik et al. 2002, Katzner et al. 2006, Karyakin et al. 2011, [Bibr B7527135][Bibr B7526779]). Reduction of their share in the species’ diet was reported for the agricultural habitat in the Ural Mnts. (Korovin 2004), as well as for the Pannonian population (Horváth et al. 2010, Horváth et al. 2018). In the European part of Turkey, the European Souslik ranked fourth with 10.65% participation (Demerdzhiev et al. 2014b).

Supporting previous findings for the EIE breeding in Kazakhstan (Katzner et al. 2005), we recorded regional diet differences. We found that dietary differences of eagles were strongly influenced by the individual territory that was occupied. The EIE preferred nesting near a high-density prey resource and used that resource almost exclusively, but in places with no such predominant high-density prey species, their diet was more diverse (Katzner et al. 2006). This circumstance was also confirmed by our study. Eagles breeding in the SG and the SP, feeding mainly on Sousliks, a colonial prey that dominated in these regions, had lower diet diversity. Similarly, eagles from the DHWstr., exploiting exclusively hedgehogs (almost 40% of their diet), also had a low diet diversity index. On the other hand, pairs in the EYP and the Sakar Mnt., with no single prevailing food resource, used diverse food. A similar dietary pattern was described for a territorial top scavenger, also hunting actively, such as the Egyptian Vulture (*Neophronpercnopterus*) (Dobrev et al. 2016). The EIE can successfully adapt to changes in the availability of prey species and utilise the most available prey sources (Demerdzhiev et al. 2014b, Katzner et al. 2005). Feeding on Storks or Hedgehogs when Sousliks are scarce is a good example of such adaptation. In the Sakar Mnt. and the neighbouring DHWstr., flocks of 50 to 250 non-breeding White Storks use the area as a foraging and roosting site during the summer (April to September) (Demerdzhiev 2011). Therefore, eagles have successfully adapted to this novel source, which is abundant in the area. A similar example was the inclusion in the eagle’s diet of various reptiles, such as Tortoises and different species of Snakes, abundant in the occupied territory and supplementing the main food source.

Our study confirmed previous findings on the EIE diet in the SG (Simeonov and Petrov 1980) identifying Sousliks as the main prey and repudiated the results of Marin et al. (2004) reported for the Sakar Mnt. and the Dervent Heights. According to Marin et al. (2004), the amount of Sousliks in the eagles’ diet from the Sakar Mnt. was very small (1.67%) and the domestic chicken was listed as the second most frequent component, accounting for 10.83%. Moreover, our study did not confirm the data assessing the European Hare (25%) as the main food source in the Dervent Heights, followed by chicken (20.83%) and European Souslik, Grey Partridge (*Perdixperdix*) and White Stork, each with an equal share (10.42%). These differences between the two studies were certainly not due to a change in the hunting behaviour of the eagles or a dramatic change in the prey availability. Marin et al. (2004) studied three nests in the Sakar Mnt. and one nest in the Dervent Heights and identified only 154 specimens of prey, findings which differ considerably from our large dataset. We highlight that this contrast can be explained by the different data collection methodology and the different volume of samples.

As we predicted, the EIE used different food resources during different seasons. While Hedgehog, Souslik and White Stork were eaten in the breeding season, in winter, the eagles fed exclusively on different rodents, mostly voles (*Microtus* sp.). However, the share of songbirds also increased significantly in the winter diet. The EIE used different foraging techniques including active hunting, kleptoparasitism or followed and foraged after tractors in agricultural fields (Danko and Mihók 2007, Zhelev et al. 2009, Demerdzhiev 2011, Horváth et al. 2018). A part of the small Passeriformes or Rodents were probably stolen from other predators or Corvids. Some of the voles and other murids were collected after land ploughing by tractors. In winter, biomass was mostly provided by carnivores and other large mammals carrion. Similar results were found for EIE overwintering in the Arabian Peninsula (Hasani et al. 2012). During this period, when food resources were limited and weather conditions were unfavourable, the EIE used various food sources and had a more diverse diet.

As with other studies (Sánchez et al. 2008, Horváth et al. 2018) concerning the diet of top predators, the inevitable limitation in our survey was related to the probability that the analysis of prey remains and/or pellets might estimate inaccurately the relative proportion of larger- (e.g. Storks, Hare) and smaller- (e.g. Vole) sized prey species, when compared to each other, caused by their different detectability. However, such large datasets indicate well the overall importance of key prey species within a region, as common prey must be detected regularly, while rare ones will be found only occasionally (Katzner et al. 2005, Horváth et al. 2018).

### Conservation implication

Our study reveals that Northern White-breasted Hedgehog, European Souslik, White Stork and European Hare were the most important prey in the diet of the EIE in Bulgaria. In summary, the species has an extremely variable diet and our work provided clear evidence of the seasonal and spatial diet diversity concerning main food sources. Availability and abundance of different prey species in the individual eagle territory determine its foraging pattern and shape diet differences. Eagles occupied territories with high-density or abundant prey exploiting exclusively that particular prey and had lower diet diversity and *vice versa* and with no single prevailing food resource, the diet was more diverse.

We found that a top predator, such as EIE, had successfully adapted to the novel food source, which was abundant in the area. The detected flexibility in the diet of the species and its ability to switch to alternative prey, if available, when the primary prey decreased, should be considered when planning species conservation efforts. If the EIE utilises a single, most abundant prey source, it depends entirely on the availability of that specific prey source (Horváth et al. 2018). This calls for the preservation of important foraging habitats, harbouring more and diverse prey, such as various mammals, birds or reptiles. In Bulgaria, a large-scale habitat alteration of the foraging places of eagles was recorded in the past decade, resulting in territory abandonment and reduced territory quality (authors data). Therefore, further research is required to better understand the links amongst habitat change, diet diversity and dietary response of the EIE.

## Supplementary Material

3A810877-A5CC-5636-99FE-A25118DD6F65 10.3897/BDJ.10.e77746.suppl1 Supplementary material 1 Brief descriptions of different taxa found in the diet of the EIE in Bulgaria Data type Table File: oo_604068.xlsx https://binary.pensoft.net/file/604068 Dimitar Demerdzhiev, Zlatozar Boev, Dobromir Dobrev, Nikolay Terziev, Nedko Nedyalkov, Stoycho Stoychev, Tseno Petrov

## Figures and Tables

**Table 1.** Distribution of localities and breeding attempts with collected food material.

**Region**	**Number of breeding sites**	**Number of breeding attempts**
Sredna Gora Mnt. (SG)	2	20
Eastern Rhodope Mnt. (ER)	1	5
Sakar Mnt.	10	126
Dervent Hights-Western foothills of Strandzha Mnt. (DHWstr.)	7	65
Elhovo-Yambol Plain (EYP)	10	58
Sliven Plain (SP)	7	32
**TOTAL**	**37**	**306**

**Table 2.** Likelihood estimation of different food categories was used to describe the regional differences in the EIE diet. Categories included in GLMM’s are given in bold.

Variable	Degrees of Freedom	Likelihood Score	p
**Lizards & Snakes**	**1**	**4.6**	**0.0 3**
**Tortoises**	**1**	**3.25**	**0.0 7**
Poultry	1	0.01	0.93
Stork	1	2.43	0.12
Raptors & Owls	1	1.85	0.17
Corvidae	1	0.74	0.39
Other birds	1	0.26	0.61
**Hedgehog**	**1**	**3.32**	**0.0 7**
Hare	1	2.35	0.12
Souslik	1	1.26	0.26
**Rodents**	**1**	**3.4**	**0.07**
Carnivores	1	0.27	0.6
Other animals	1	0.27	0.6

**Table 3.** List of GLMMs used for the analysis of EIE diet; Food components in their participation as a prey item (A) and biomass contribution (B) were presented. All models with ∆AIC < 2 were considered best models; model weight value (*w)*; RI – relative importance value of each of the candidate models; Parameter estimates ± SE, Lower (95%) and Upper CL (95%) of explanatory factors, their importance value (Wald Stat.) and a probability value (p) were taken from the average model.

**N**	**Model structure (A)**	**AIC**	**ΔAIC**	***w***	**RI**	**p**
1	Random effect	195.24	0.00	0.46	1	0.005
2	Hedgehog	197.04	1.80	0.19	0.41	0.01
3	Tortoises	197.11	1.87	0.18	0.39	0.01
4	Lizards & Snakes	197.22	1.98	0.17	0.37	0.01
**N**	**Model structure (B)**	**AIC**	**ΔAIC**	***w***	**RI**	**p**
1	Random effect	195.24	0.00	0.57	1	0.005
2	Lizards & Snakes	197.15	1.91	0.22	0.39	0.01
3	Rodents	197.24	2.00	0.21	0.37	0.01
**N**	**Explanatory variables**	**Estimate**	**St. err.**	**Wald Stat.**	**Lower CL/Upper CL**	**p**
1	Random effect	4.72	0.11	1824.78	4.51 / 4.94	< 0.001
2	Hedgehog	0.01	0.02	0.19	-0.02 / 0.04	0.66
3	Tortoises	0.01	0.02	0.13	-0.03 / 0.04	0.72
4	Lizards & Snakes	0.0005	0.03	0.0001	-0.06 / 0.06	0.99
5	Rodents	-0.003	0.05	0.003	-0.1 / 0.1	0.96
